# Prevention of incisional hernia at the site of stoma closure with different reinforcing mesh types: a systematic review and meta-analysis

**DOI:** 10.1007/s10029-021-02393-w

**Published:** 2021-03-13

**Authors:** Roberto Peltrini, Nicola Imperatore, Gaia Altieri, Simone Castiglioni, Maria Michela Di Nuzzo, Luciano Grimaldi, Michele D’Ambra, Ruggero Lionetti, Umberto Bracale, Francesco Corcione

**Affiliations:** 1 Department of Public Health, University of Naples Federico II, Naples, Italy; 2 Department of Clinical Medicine and Surgery, University of Naples Federico II, Naples, Italy; 3 Gastroenterology and Endoscopy Unit, AORN Antonio Cardarelli, Naples, Italy; 4 Departement of Gastroenterological, Endocrine-Metabolic and Nephrourological Sciences, Università Cattolica del Sacro Cuore, Rome, Italy; 5 Department of Medical, Oral and Biotechnological Sciences, University G. D’Annunzio Chieti-Pescara, Chieti, Italy

**Keywords:** Stoma reversal, Incisional hernia, Biologic mesh, Synthetic mesh, Prevention

## Abstract

**Purpose:**

To evaluate safety and efficacy of a mesh reinforcement following stoma reversal to prevent stoma site incisional hernia (SSIH) and differences across the prostheses used.

**Methods:**

A systematic search of PubMed/MEDLINE, EMBASE, SCOPUS and Cochrane databases was conducted to identify comparative studies until September 2020. A meta-analysis of postoperative outcomes and a network meta-analysis for a multiple comparison of the prostheses with each other were performed.

**Results:**

Seven studies were included in the analysis (78.4% ileostomy and 21.6% colostomy) with a total of 1716 patients with (*n* = 684) or without (*n* = 1032) mesh. Mesh placement was associated with lower risk of SSIH (7.8%vs18.1%, OR0.266,95% CI 0.123–0.577, *p* < 0.001) than no mesh procedures but also with a longer operative time (SMD 0.941, 95% CI 0.462–1.421, *p* < 0.001). There was no statistically significant difference in terms of Surgical Site infection (11.5% vs 11.1%, OR 1.074, 95% CI 0.78–1.48,* p* = 0.66), seroma formation (4.4% vs 7.1%, OR 1.052, 95% CI 0.64–1.73,* p* = 0.84), anastomotic leakage (3.7% vs 2.7%, OR 1.598, 95% CI 0.846–3.019,* p* = 0.149) and length of stay (SMD − 0.579,95% CI − 1.261 to 0.102,* p* = 0.096) between mesh and no mesh groups. Use of prosthesis was associated with a significant lower need for a reoperation than no mesh group (8.1% vs 12.1%, OR 0.332, 95% CI 0.119–0.930,* p* = 0.036). Incidence of seroma is lower with biologic than polypropylene meshes but they showed a trend towards poor results compared with polypropylene or biosynthetic meshes.

**Conclusion:**

Despite longer operative time, mesh prophylactic reinforcement at the site of stoma seems a safe and effective procedure with lower incidence of SSIH, need for reoperation and comparable short-term outcomes than standard closure technique. A significant superiority of a specific mesh type was not identified.

**Supplementary Information:**

The online version contains supplementary material available at 10.1007/s10029-021-02393-w.

## Introduction

A defunctioning stoma is often performed after low anterior resection with Total Mesorectal excision (TME) for rectal cancer to decrease the risk of symptomatic anastomotic leakage [1–3]. Restoration of intestinal continuity is routinely planned 6–8 weeks after rectal surgery. Despite considered as a relative safe procedure, many studies reported high morbidity rates following loop ileostomy closure with different types of complication and underestimated consequences [4, 5].

Stoma site incisional hernia (SSIH) is associated with significative long-term morbidity, causing pain, deformity, and obstruction and requiring reoperation in most of the patients. Additionally, incisional hernia has significant impact on health-related quality of life and body image [6]. Incidence of SSIH varies from 0 to 40% with a great heterogeneity across studies and higher rates for colostomies than ileostomies [7, 8]. In particular, the pooled estimate proportion of incisional hernia was 6% after loop ileostomy reversal reaching 13% when only the studies designed with incisional hernia detection as a primary outcome were considered [9]. The rate of SSIH increases when imaging was used for diagnosis rather than clinical evaluation [7, 9].

Mesh placement demonstrated to significantly reduce the incidence of incisional hernia after midline laparotomy compared with primary suture [10, 11]. Thus, a prophylactic mesh strategy was adopted to reinforce abdominal wall at the site of stoma reversal [12]. However, this preventive measure had to consider some concerns when adopted to ileostomy reversal, such as defect location away from midline and the risk of mesh infection. In fact, bowel content contamination at the stoma closure site is inevitable, increasing risk of wound infections and impairing healing process [13]. The historical dogma of the contraindication of permanent prostheses in contaminated surgical fields [14, 15] seemed to have been overcome by the use of resorbable meshes [16]. To date, three different types of mesh are available in clinical practice: synthetic non-absorbable, synthetic absorbable (biosynthetic) and biologic meshes [17]. All of them were used to prevent incisional hernia after stoma closure; however, there is still no consensus on the type of prosthesis and the placement technique to be adopted.

The aim of this review is to evaluate the safety and efficacy of a mesh prophylactic strategy following stoma reversal. Additionally, we aimed to compare postoperative outcomes of each specific mesh type through network meta-analysis to identify the superiority and the differences of one prosthesis over the others.

## Materials and methods

### Literature search and selection of primary studies

The strategy for building the evidence base for the assessment of the outcomes of stoma closure with or without prophylactic mesh reinforcement was performed with a systematic review of the existing evidence in the literature, conducted in accordance with the preferred reporting items for systematic reviews and meta-analyses (PRISMA) guidelines [18].

The systematic literature review was performed in PubMed/MEDLINE, EMBASE, SCOPUS and Cochrane databases to identify studies that compared outcomes of stoma closure with or without mesh reinforcement from the beginning of indexing for each database till September 1, 2020. Bibliographic review of selected articles was assessed as secondary sources for full-length articles of studies. A literature search was performed and verified by 2 independent reviewers (R.P. and N.I.) using the following index terms: “stoma closure” AND “mesh” OR “ileostomy” AND “mesh” OR “colostomy” AND “mesh” OR “prophylactic mesh” OR “stoma reinforcement”.

### Eligibility criteria

Two reviewers (R.P. and N.I.) independently evaluated all the studies retrieved according to the eligibility criteria and any differences between the datasets were resolved by discussion. Studies were included if they met all of the following criteria: (1) randomized controlled trial (RCT), prospective or retrospective studies comparing stoma closure with or without mesh reinforcement; (2) original studies published in a peer-reviewed journal; (3) studies involving adult patients (aged > 18 years). We excluded the articles if there was no sufficient documentation on—or no possibility to calculate—the percentage of SSIH (primary endpoint), if they were in languages other than English, if they were focused on pediatric patients. Narrative reviews, duplicate publications and editorials were also excluded.

### Data extraction and management

Data were extracted independently and entered into standardized Excel spreadsheets (Microsoft Inc., Redmond, Washington, USA). Any disagreements were resolved through discussion. The following data were extracted from each study: first author, year of publication, study design, sample size, stoma type (ileostomy or colostomy), type of mesh used (biologic, biosynthetic, polypropylene), site of mesh placement (Retromuscular, Intrabdominal, Onlay), number of subjects developing SSIH in mesh and no mesh group, operative time (minutes), number of patients developing surgical site infections (SSI) in both groups, percentage of seroma formation, number of anastomotic leaks, need for reoperation, and length of stay (days) in both groups.

Primary study outcome was the assessment of SSIH development in the two groups (mesh vs no mesh) at the end of follow-up for each included study. Furthermore, secondary outcomes included the evaluation of differences in: operative time, SSI development, seroma formation, anastomotic leak, need for reoperation and length of stay.

### Statistical analysis

Statistical analyses were performed using Comprehensive Meta-analysis Software version 3.0 (Biostat, Englewood, New Jersey, USA).

Heterogeneity was assessed using chi-squared statistics and I2 measure of inconsistency. The quality of the analyzed studies and publication bias was evaluated by two reviewers (R.P. and N.I) in consensus using a quality assessment tool for diagnostic accuracy studies (QUADAS-2) [19]. The risk of publication bias and concerns regarding the applicability of studies were then assessed by visually inspecting QUADAS-2 plots.

The meta-analysis was conducted using a fixed-effect model in the case of non-significant heterogeneity (*p* > 0.1), and a random effect model (DerSimonian–Laird method) when significant heterogeneity was present (*p* < 0.1). Corresponding forest plots were constructed for the pooled estimates of these outcomes and weight of individual studies are represented by the size of individual squares. The odds ratio (OR) was assessed for dichotomous outcomes, while standardized mean difference (SMD) with 95% confidence interval (CI) was estimated for continuous outcomes.

Furthermore, a random effect meta-regression was performed to evaluate possible patient (age, gender, comorbidities such as diabetes, BMI, respiratory diseases) or disease (reason for stoma creation) or technical (stoma type, site of mesh placement) variables able to impact upon the outcomes.

Finally, we conducted a network meta-analysis to compare the different types of mesh (biologic, polypropylene and biosynthetic) on the risk of SSIH, SSI, reoperation, seroma formation and anastomotic leak using a multivariate random-effects meta-regression. We used a frequentist approach based on a random-effects consistency model and provided a point estimate from the network along with 95% CI from the frequency distribution of the estimate.

A *p* value < 0.05 was considered statistically significant for all outcomes.

## Results

**Fig. 1:**
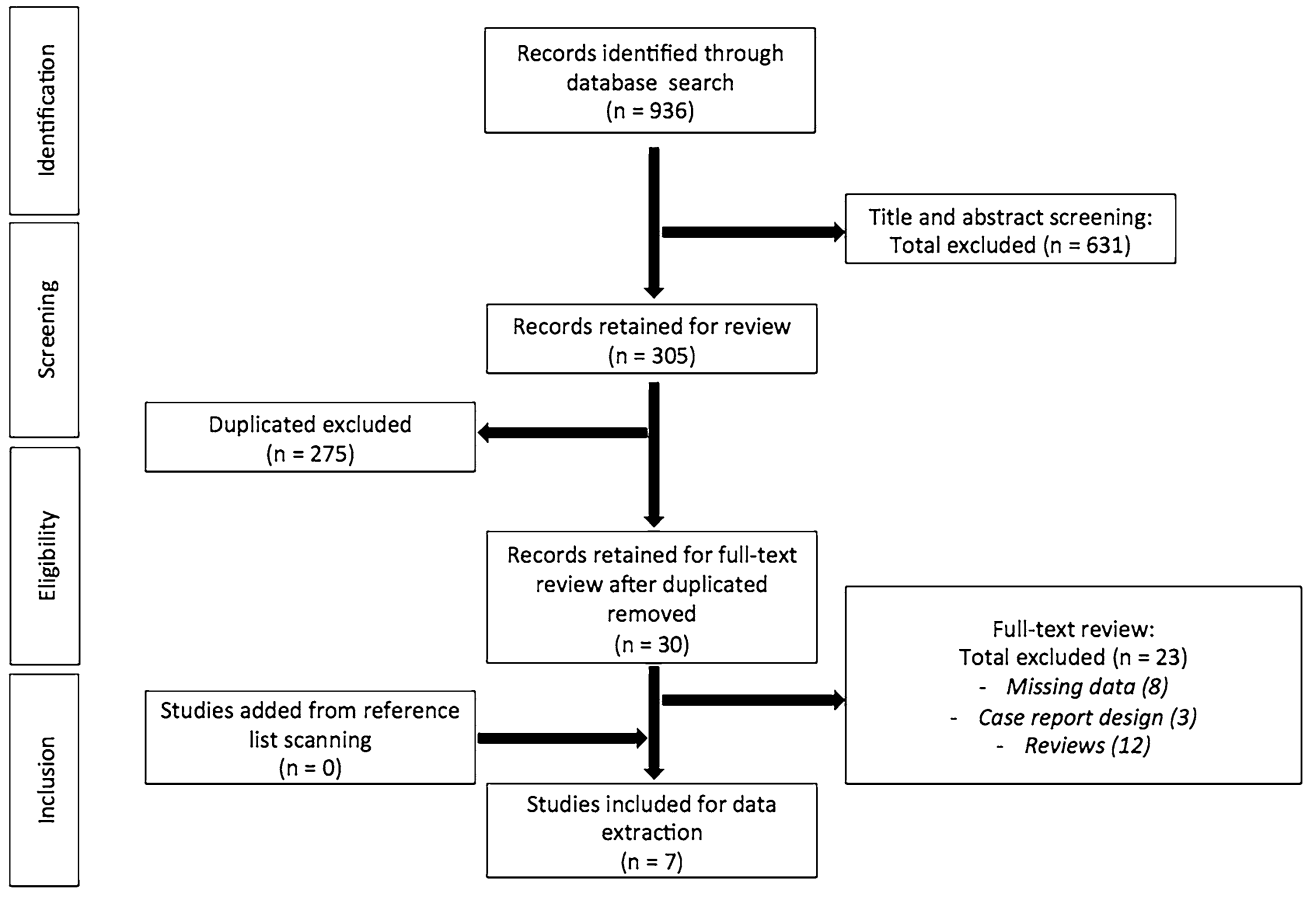
Flow diagram of the search strategy and selection of studies included in the meta-analysis

Figure 1 shows the PRISMA flow diagram of the literature selection process. The search strategy identified a total of 936 publications in the initial search. After the screening of title and abstract and removal of duplicates, 30 articles were selected for further review. After exclusion of 23 articles, 7 studies were included in the meta-analysis [20–26]. In accordance with the inclusion criteria, one study was a RCT [22], while the remaining were retrospective studies [20, 21, 23–26]. Moreover, while five studies [20, 23–26] involved patients undergoing ileostomies, two studies [21, 22] enrolled both ileostomies and colostomies.

Furthermore, three studies used a biologic mesh [20, 22, 26], three studies used a polypropylene mesh [21, 23, 25] and one study used a biosynthetic mesh [24]. The mesh placement was retromuscular in two studies [20, 21], onlay in four studies [23–26] and intrabdominal in the remaining one [22].

Finally, a total of 1716 patients who underwent stoma closure (78.4% ileostomy and 21.6% colostomy) with (*n* = 684) or without (*n* = 1032) were included in the meta-analysis. Table 1 shows the characteristics of the studies included.

**Table 1:** Details of studies selected for meta-analysis

References	Study design	Sample size	Stoma type	Type of Mesh	Mesh Placement	Study group	*N* patient
Maggiori 2015 [20]	Retro	94	Ileostomy (All)	Biologic	Sublay	Mesh	30
						No Mesh	64
Warren 2017 [21]	Retro	359	Ileostomy (147) Colostomy (212)	Polypropylene	Sublay	Mesh	91
						No Mesh	268
Bhangu 2020 [22]	RCT	790	Ileostomy (631) Colostomy (159)	Biologic	IPOM	Mesh	394
						No Mesh	396
Wong 2020 [23]	Retro	273	Ileostomy (All)	Polypropylene	Onlay	Mesh	81
						No Mesh	192
Pizza 2020 [24]	Retro	84	Ileostomy (All)	Biosynthetic	Onlay	Mesh	26
						No Mesh	58
Liu 2013 [25]	Retro	83	Ileostomy (All)	Polypropylene	Onlay	Mesh	47
						No Mesh	36
Lee 2020 [26]	Retro	33	Ileostomy (All)	Biologic	Onlay	Mesh	15
						No Mesh	18

*RCT* randomized clinical trial, *SSIH* stoma site incisional hernia, *SSI* surgical site infection, *IPOM* open intraperitoneal onlay mesh

### Quality of studies and risk of bias

The studies showed a low-to-moderate risk of bias and a few concerns about applicability. Five studies scored low risk of bias in all domains of the QUADAS-2 system. The highest risk of bias was associated to flow and timing. Considering concerns regarding applicability, all studies but one presented a low risk.

### Stoma site incisional hernia

All studies reported the development of SSIH, with an overall rate of 12.9%.

**Fig. 2:**
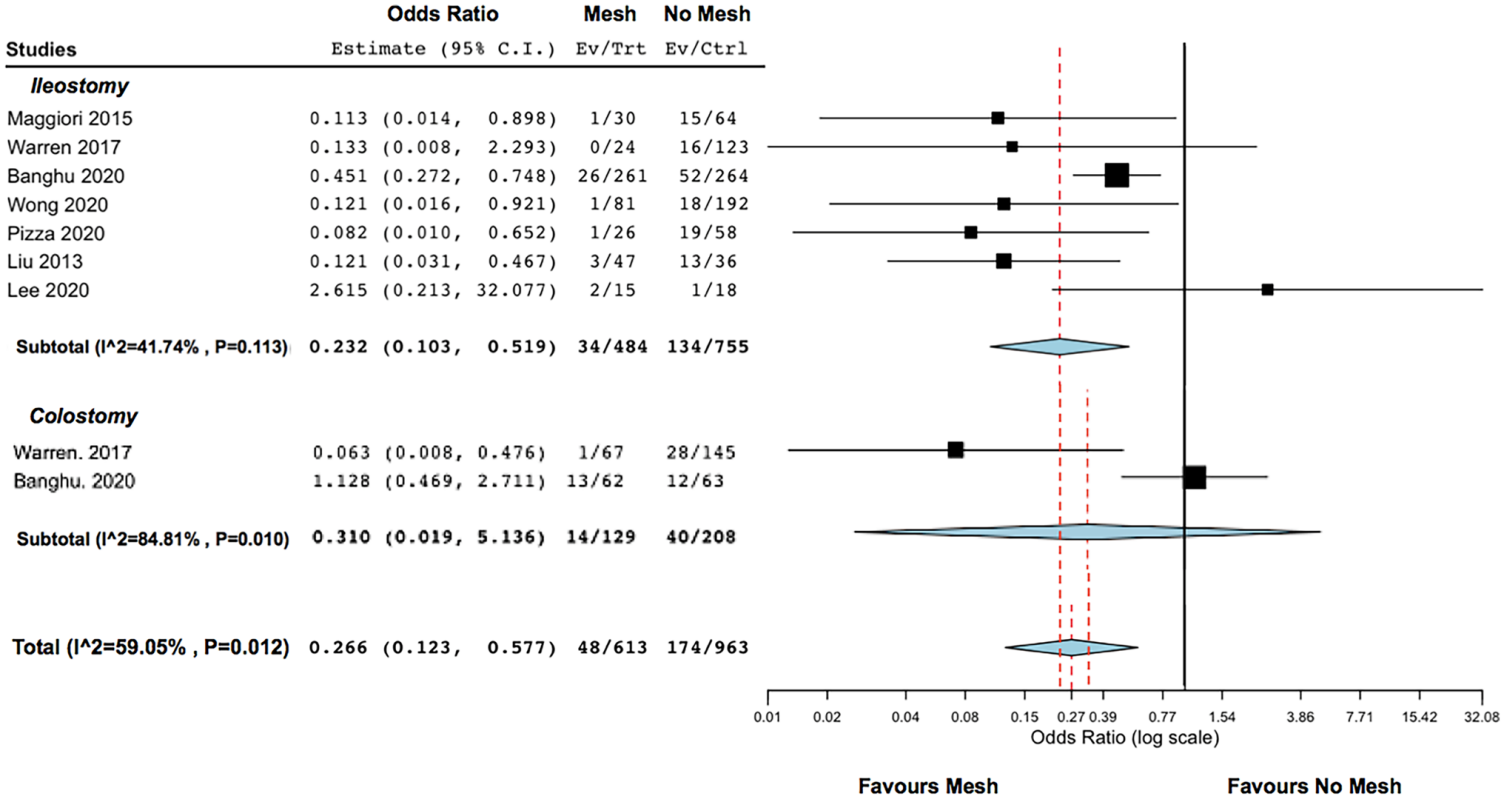
Forest plots of outcomes included in the analysis: SSIH

Stoma closure with mesh placement was associated with lower risk of SSIH (7.8% vs 18.1%, OR 0.266, 95% CI 0.123–0.577, *p* < 0.001) than no mesh procedures (Fig. 2). This analysis presented high heterogeneity (I2 = 59.1%, *p* = 0.01).

**Fig. 3:**
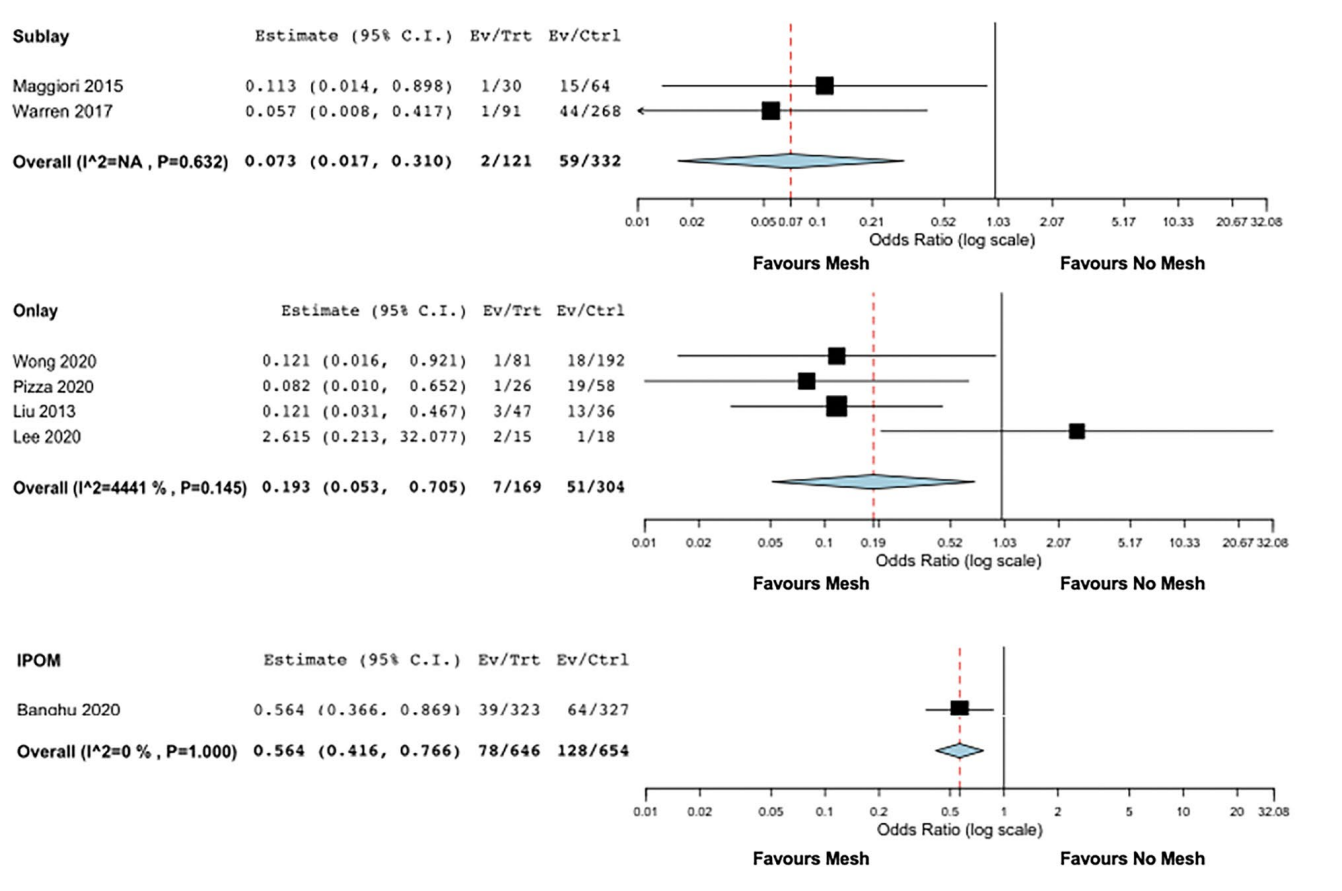
Forest plots of outcomes included in the analysis: SSIH and techniques (onlay, sublay, IPOM)

In the ileostomy subgroup (*n* = 1345), the rate of SSIH was 12.4% and it was significantly lower in patients undergoing stoma closure with mesh (7% vs 17.7%, OR 0.232, 95% CI 0.103–0.519, *p* < 0.001) (Fig. 3a). No heterogeneity was found (I2 = 41.7%, *p* = 0.11).

In the colostomy group (*n* = 371), the rate of SSIH was 13.7% but there was no significant difference in SSIH development between groups (10.8% in the mesh group vs 19.2% in the no mesh group, OR 0.310, 95% CI 0.019–5.136, *p* = 0.41) (Fig. 2). High heterogeneity was found in this analysis (I2 = 84.8%, *p* = 0.01).

We performed further sub-analyses to establish the role of technique and time of radiological assessment. Despite the absence of studies directly comparing different techniques (sublay vs onlay vs intrabdominal), we found a significantly lower rate of SSIH in patients undergoing stoma closure with mesh, independently from technique used (sublay OR 0.073, 95% CI 0.017–0.31; onlay OR 0.193, 95% CI 0.053–0.705; IPOM OR 0.564, 95% CI 0.416–0.766, see Fig. 3). Moreover, although four studies reported a longer follow-up in the no mesh group (with a significant difference reported only in two studies [21, 24]), this meta-analysis did not demonstrate any difference in the length of follow-up between mesh and no mesh group (Supplementa material S1).

Then, we performed a meta-regression to evaluate possible patient (age, gender, comorbidities such as diabetes, BMI, respiratory diseases) or disease (reason for stoma creation) or technical (stoma type, site of mesh placement) variables able to impact upon the outcomes.

At meta-regression, no variable impacted the SSIH development (age *p* = 0.4; gender male vs female *p* = 0.33; diabetes *p* = 0.2; BMI *p* = 0.15; respiratory diseases *p* = 0.34; reason for operation malignancy vs no malignancy *p* = 0.31; ileostomy vs colostomy *p* = 0.5, retromuscular vs intrabdominal *p* = 0.23, retromuscular vs onlay *p* = 0.61, intrabdominal vs onlay *p* = 0.31).

### Operative time

We found 4 studies [21, 22, 24, 26] involving 1266 patients, reporting operative time differences between mesh and no mesh groups. A longer operative time was reported in the mesh group when compared with no mesh group, (SMD 0.941, 95% CI 0.462–1.421, *p* < 0.001). High heterogeneity was found (I2 = 88.95%, *p* < 0.001).

### Surgical site infection

All studies reported the rate of SSI. The cumulative rate was therefore 11.3%. There was no statistically significant difference in terms of risk of SSI development between mesh and no mesh groups (11.5% vs 11.1%, OR 1.074, 95% CI 0.78–1.48, *p* = 0.66) (Supplemental material S2). Also, in this case, there was no heterogeneity (I2 = 0%, *p* = 0.87).

### Seroma formation

The development of seroma was reported in 6 studies [20–24, 26] involving 1633 subjects. Stoma closure with mesh positioning was not associated with higher risk of seroma formation than no mesh (4.4% vs 7.1%, OR 1.052, 95% CI 0.64–1.73, *p* = 0.84). Heterogeneity was very low (I2 = 0%, *p* = 0.84).

### Anastomotic leak

Four studies [20–22, 24] reported the rate of anastomotic leak in the two groups on a total of 1327 subjects. There was no significant difference in terms of risk of anastomotic leak development between mesh and no mesh groups (3.7% vs 2.7%, OR 1.598, 95% CI 0.846–3.019, *p* = 0.149).

Overall heterogeneity was low in this analysis (I2 = 0%, *p* = 0.65).

### Need for a second operation

The need for reoperation was reported by five studies [20, 22–25] and 1324 patients. Stoma closure with mesh reinforcement was associated with a significant lower need for a second operation than no mesh group (8.1% vs 12.1%, OR 0.332, 95% CI 0.119–0.930, *p* = 0.036) (Supplemental material S3). No heterogeneity was found (I2 = 33.5%, *p* = 0.2).

### Length of hospital stay

The length of stay was reported by five studies [20, 21, 24–26] and 653 patients. Stoma closure with mesh was not associated with a significant longer hospital stay than no mesh group (SMD − 0.579, 95% CI − 1.261 to 0.102, *p* = 0.096). Overall heterogeneity was high in this analysis (I2 = 91.9%, *p* < 0.001).

### Network meta-analysis

Combining all the indirect comparisons in a network meta-analysis to compare different mesh types with each other, we did not identify a significant superiority of a specific mesh type in reducing the risk of SSIH, SSI, need for a second operation and anastomotic leak.

Specifically, as compared with polypropylene and biosynthetic meshes, biologic mesh was associated with higher probability of SSIH development (OR 1.76, 95% CI 0.94–4.28 and OR 1.23, 95% CI 0.66–3.91, respectively), SSI development (OR 1.57, 95% CI 0.87–3.15 and OR 1.46, 95% CI 0.63–4.56, respectively), risk of a second operation (OR 1.93, 95% CI 0.92–6.37 and OR 1.61, 95% CI 1.61, 95% CI 0.51–3.41, respectively), risk of anastomotic leak (OR 1.34, 95% CI 0.67–3.21 and OR 1.11, 95% CI 0.48–2.43, respectively), although all these results were not significant. Instead, biologic mesh was associated with significant lower risk of seroma development than polypropylene mesh (OR 0.65, 95% CI 0.26–0.94). No significant differences were found between biologic and biosynthetic meshes (OR 0.91, 95% CI 0.58–2.14) and between polypropylene and biosynthetic meshes (OR 1.67, 95% CI 0.76–2.37) in terms of seroma formation. Table 2 shows detailed results of network meta-analysis.

**Table 2:** Network meta-analysis comparing different types of meshes

	OR (95% CI)		
	Biologic	Polypropylene	Biosynthetic
A. Network meta-analysis comparing type of Mesh and risk of SSIH			
Biologic	–		
Polypropylene	1.76 (0.94–4.28)	–	
Biosynthetic	1.23 (0.66–3.91)	0.85 (0.34–2.02)	–
B. Network meta-analysis comparing type of Mesh and risk of SSI			
Biologic	–		
Polypropylene	1.57 (0.87–3.15)	–	
Biosynthetic	1.46 (0.63–4.56)	1.21 (0.32–3.32)	–
C. Network meta-analysis comparing type of Mesh and risk of a second operation			
Biologic	–		
Polypropylene	1.93 (0.92–6.37)	–	
Biosynthetic	1.61 (0.51–3.41)	0.76 (0.32–2.13)	–
D. Network meta-analysis comparing type of Mesh and risk of seroma			
Biologic	–		
Polypropylene	**0.65 (0.26–0.94)**	–	
Biosynthetic	0.91 (0.58–2.14)	1.67 (0.76–2.37)	–
E. Network meta-analysis comparing type of Mesh and risk of anastomotic leak			
Biologic	–		
Polypropylene	1.34 (0.67–3.21)	–	
Biosynthetic	1.11 (0.48–2.43)	0.87 (0.47–2.75)	–

Comparisons should be read from left to right and from up to down. Statistically significant results are expressed in bold

## Discussion

Prophylactic mesh reinforcement at the site of stoma reversal is an effective procedure to reduce postoperative SSIH and it is not associated with higher incidence of SSI, seroma and anastomotic leakage than a control group, although a longer operative time for the procedure. In this meta-analysis the overall rate of SSIH is 12.9%. This value is in accordance with previous reports in which the incidence varies between 6.5 and 30% [7, 8]. Furthermore, need for a reintervention is significantly more frequent in no mesh group, strengthening the efficacy of a strategy for the use of a prophylactic mesh. By all possible indirect comparison, none of the three types of prostheses seems to give significantly better results than the others to date, except for a lower incidence of seroma formation of biologic compared to polypropylene meshes.

These results are similar to those of two recent meta-analysis [12, 27]. However, this is the most up-to-date and comprehensive review which aimed to investigate, not only safety and effectiveness of mesh prophylactic strategy, but also the potential benefits or drawbacks of each specific mesh type versus the others by comparing postoperative outcomes. Although no significant difference emerged in the incidence of SSIH by using different mesh types, some important considerations can be drawn from the analysis. Additionally, we emphasize that the number of studies on the subject from the first review [12] has more than doubled in about one year highlighting the efforts in advancing of incisional hernia prevention.

The use of prosthetic material has found wide application in surgery [10, 28, 29]. While it seems that mesh use during permanent end colostomy construction cannot help to reduce parastomal hernia from recent RCT [28, 30], prophylactic mesh strategy to prevent SSIH following stoma reversal has been explored only in recent years with the remarkable results of the ROCCS trial [22] that certainly may influence current clinical practice.

The included studies in the present review reported differences in terms of mesh type and surgical techniques concerning mesh location with onlay, sublay and open intraperitoneal onlay mesh (IPOM) repair. This means that there is still not a consensus on the optimal management for mesh placement at the site of stoma closure. The PRIMA trial [31] evaluated the effectiveness of mesh reinforcement in high-risk patients after midline laparotomy to prevent incisional hernia. Incisional hernia rate differed significantly between onlay mesh reinforcement and primary suture, but it did not differ comparing sublay mesh reinforcement versus primary suture or onlay versus sublay mesh reinforcement. Therefore, authors argue a stronger and more significant effect on prevention of incisional hernia of onlay than sublay mesh reinforcement. However, in incisional hernia repair, onlay is associated with markedly more wound complications and seroma rates [32] and this is to bear in mind when a contaminated operation such as stoma reversal is performed.

In contaminated surgical fields, using biologic mesh derived from the collagen-rich tissues of human, porcine, or bovine sources [33] seems the obvious option. However, evidence does not support the superiority of resorbable over non-resorbable meshes in ventral hernia repair under contaminated conditions [34–36]. Biologic mesh integration, remodeling and reabsorption by the host certainly affect tensile strength and resistance to infection with implications on their use [37]. In fact, disappointing clinical outcomes have been achieved in some studies concerning use of biologic mesh for abdominal wall defects [38–41] such as the results of the present meta-analysis found a trend toward worse short- and long-term outcomes when compared with polypropylene and biosynthetic meshes, although all these results were not statistically significant. However, in the present study, it is significant a lower rate of postoperative seroma with use of biologic than polypropylene mesh.

The choice not to use a synthetic non-resorbable prosthesis is reasonably acceptable due to the high risk of local infection complications in a site with intestinal bacterial contamination [42]. By contrast, the included studies in the present review which used polypropylene meshes [21, 23, 25] did not report higher SSI or wound infection rates than no mesh control group.

It is reasonable to consider that biosynthetic prostheses may represent a fair compromise since they were developed as a possible cost-effective alternative to the biologic meshes [34] sharing their tolerance in contaminated fields and the tensile strength of synthetic meshes.

Biosynthetic meshes demonstrated a clinical effectiveness in complex ventral hernia repair [43–45] with lower complication and reherniation rates when compared to biologic meshes [46]. Furthermore, in contaminated ventral hernia repair, the COBRA study [47] reported the overall hernia recurrence rate was 17% at 24 months using biosynthetic meshes, lower than in a similar designed study with biologic meshes (28%) [48]. However, in the present review only one study refers to biosynthetic meshes [24] and the superiority of a specific prosthesis over others remains to be demonstrated in this setting.

The current study has some limitations. Only one RCT was included and only one more retrospective study was added than others review [27] with a limited number of patients. There is a great heterogeneity among studies regarding patients’ population, mesh placement, type of prosthesis and fascial closure technique in the control group. Finally, there are not comparative studies evaluating differences in the use of two different meshes and only in one of them biosynthetic meshes were used. The absence of direct comparison does not allow definitive conclusions to be drawn. However, considering the recent and rapid developments of mesh prophylactic strategy during stoma closure, this study provides a comprehensive overview on the subject with implications in current clinical practice. Lower SSIH incidence and need for second operation along with a comparable postoperative complication rate than a control group would confirm safety and effectiveness of mesh reinforcement at the stoma site. The most frequent mesh-related concerns limiting their use in a contaminated surgical field seem averted, but many aspects remain to be explored. This study aims to take a first step towards identifying the best performing prosthesis to be applied at the stoma site. Although no significative difference in terms of hernia occurrence and morbidity we noted a worse trend of biologic meshes in this setting which is in accordance with some reports from the literature.

While the effectiveness of a mesh prophylaxis strategy to reduce the incidence of parastomal hernia after permanent colostomy construction has been questioned by recent RCTs [28, 30], mesh placement at site stoma reversal is a relatively more recent and still debated issue. Only one multicenter double-blind RCT was included in the analysis, along with single-center retrospective studies. Therefore, we are unable to recommend routine mesh use during ostomy closure, despite the encouraging results on its preventive role and safety.

Need for prosthetic material during surgery is always carefully evaluated because of the potential mesh-related complications. This may affect mesh use aiming to prevent and a complication, espacially in contamineted surgical field. However, patients with incisional hernia experience a lower health-related quality of life on physical components and worse body image [6] and they are often reluctant to a further operation and more challenging to treat than those with primary hernia [49]. Therefore, we believe that patient selection is the most suitable compromise in the light of current evidence. We suggest an accurate assessment of risk factors of SSIH such as male gender, high BMI, concomitant diseases and presence of a midline incisional hernia [50–52]. According to Fischer’s risk model and stratification system, stoma reversal is considered an independent risk factor for surgically treated incisional hernia [53]. Developing a scoring system tool to predict SSIH could improve preoperative risk assessment and direct towards the optimal surgical strategy such as for ventral incisional hernia [54].

Although we found that SSIH rate is independent from surgical technique, it is reasonable that retromuscular plane can be considered the optimal mesh location, away from skin and subcutaneous contaminated tissue avoiding bowel contact. Finally, when peritonitis occurs because of anastomosis breakdown, mesh can be removed to re-establish a diverting stoma if required.

Further studies focusing on mesh placement in relation to fascial layers can give a contribute to standardize the most appropriate surgical technique, in this setting not only from high specialization centers [55]. Likewise, comparative analysis between mesh type to use [56] would address the surgeons towards a safer, more efficacy and cost-effective choice.

## Conclusion

Evidence in favor of prophylactic meshes to prevent incisional hernias following stoma reversal is rising. Despite longer operative time, mesh prophylactic reinforcement at the site of stoma seems a safe and effective procedure with lower incidence of SSIH, need for reoperation and comparable short-term outcomes than standard closure technique. No significant differences were found among mesh types, but a trend towards poorer results was recorded for biologic meshes.

Future studies should directly compare outcomes of different meshes and investigate the most appropriate surgical placement technique to prevent SSIH.

## Supplementary Information

Below is the link to the electronic supplementary material.

Supplementary file1 (DOCX 344 KB)
