# Supplementary material for: Prevention of incisional hernia at the site of stoma closure with different reinforcing mesh types: a systematic review and meta-analysis
Source: Hernia. 2021 Mar 13;25(3):639–48. doi: 10.1007/s10029-021-02393-w (PMC8197707; doi:10.1007/s10029-021-02393-w)

**Supplemental material**

**S1** Forest plots of outcomes included in the analysis: SSIH and length of follow-up


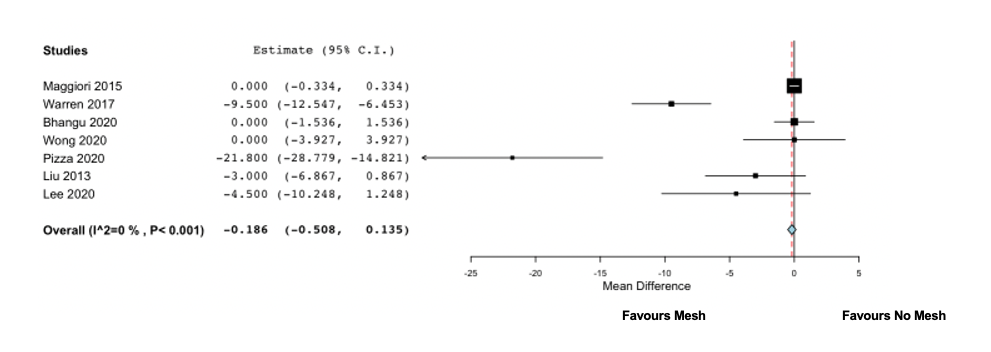


**S2** Forest plots of outcomes included in the analysis: SSI (surgical site infection)


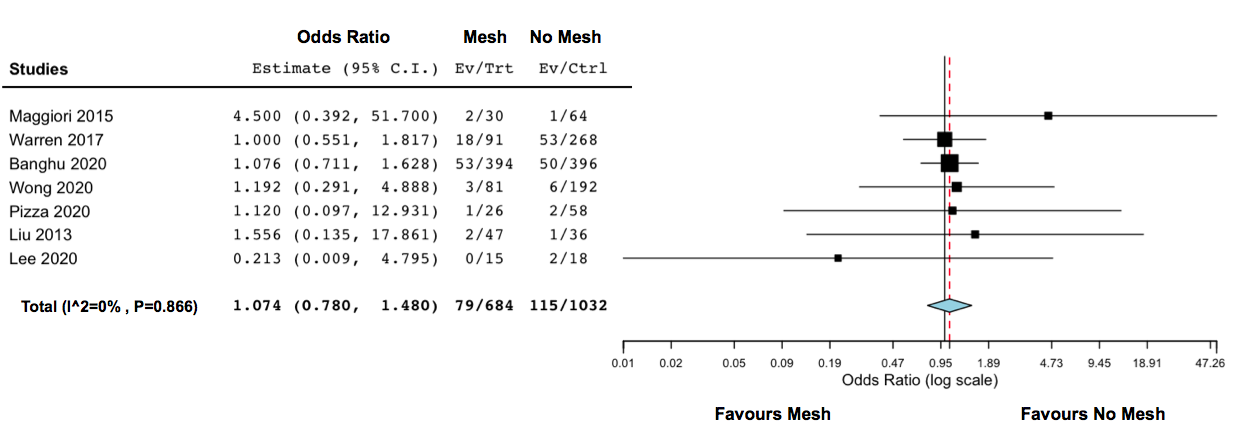


**S3** Forest plots of outcomes included in the analysis: Need for second operation


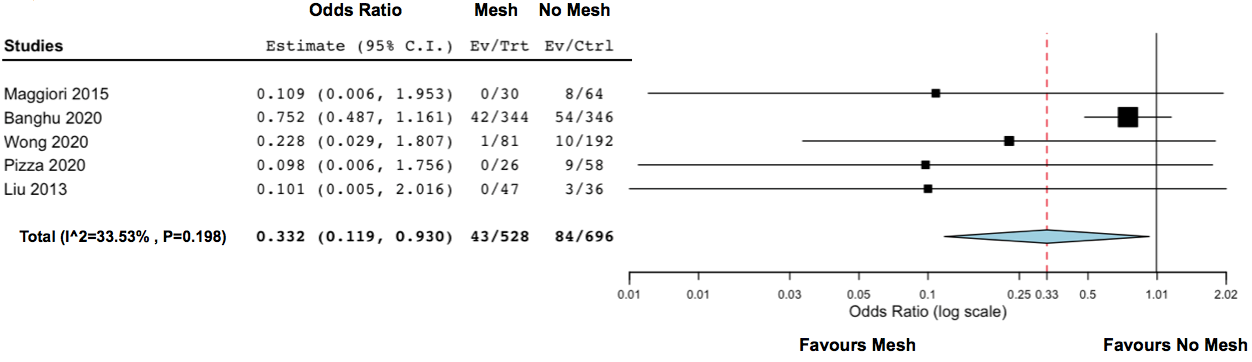

Supplement: Supplementary file 1 — Supplementary file1 (DOCX 344 KB) [file 10029_2021_2393_MOESM1_ESM.docx]
